# Trends in Incidence Rates of Tobacco-Related Cancer, Selected Areas, SEER Program, United States, 1992-2004

**Published:** 2008-12-15

**Authors:** Anthony P. Polednak

**Affiliations:** Connecticut Department of Public Health

## Abstract

**Introduction:**

Recent trends in incidence rates for tobacco-related cancers may vary geographically because of variation in socioeconomic status and in history of comprehensive state tobacco control programs (starting with California in 1989). Recent trends in risk factors are likely to affect cancer incidence rates at the youngest ages.

**Methods:**

Trends in age-adjusted incidence rates for cancers most strongly associated with tobacco (ie, lung, oral cavity-pharynx, and bladder cancers) were analyzed for 1992 through 2004 in 11 areas (the states of Connecticut, Hawaii, Iowa, Utah, and New Mexico, and the metropolitan areas of Atlanta, Georgia; Detroit, Michigan; Los Angeles County, California; San Francisco-Oakland, California; San Jose-Monterey, California; and Seattle-Puget Sound, Washington) in the Surveillance, Epidemiology and End Results (SEER) Program. The 8 states differed in poverty rate of the population and in history of statewide tobacco control efforts as measured by an initial outcomes index (IOI) for the 1990s and a strength of tobacco control (SoTC) index for 1999 through 2000. Annual percentage change (APC) in incidence rate was calculated for whites and blacks separately and by sex for each SEER area.

**Results:**

Among whites, the largest declines for lung cancer were in the 3 SEER areas of California, which were the only areas with significant (negative) APCs for oral cavity-pharynx cancer (but not for bladder cancer). For blacks, significant (negative) APCs for both lung and oral cavity-pharynx cancers were found in 4 of 5 areas with useful data but only 1 of 3 areas for bladder cancer. The strongest correlations of APCs for whites were for lung and oral cavity-pharynx cancers with the IOIs for the early 1990s and with the SoTC (due to the influence of California, which had the highest SoTC).

**Conclusion:**

Lung and oral cavity-pharynx cancer incidence rates among whites aged 15 to 54 years declined more in California than in other areas, possibly because of comprehensive state tobacco control efforts. The different trends for bladder cancer vs other cancers could reflect the influence of risk factors other than tobacco. The greater geographic uniformity of trends among blacks than among whites for lung and oral cavity-pharynx cancers requires further study, particularly in relation to state tobacco control efforts.

## Introduction

Tobacco control has been addressed in many comprehensive cancer control plans, but efforts must be expanded (1). California had the earliest (1989) statewide comprehensive tobacco control program, whereas several southern tobacco-growing states have had limited efforts (1-3). Temporal trends in cancer incidence rates among younger adults may be indicators of recent trends (eg, in cancer control efforts) that may affect prevalence of cancer risk factors, whereas rates for older populations are also affected by events in the distant past (4). Trends in lung cancer mortality (5) and incidence (6) rates in various US states among young adults have been used in assessing the potential effect of state tobacco control efforts, as measured by a state "initial outcomes index" or IOI (2) (also called "index of tobacco control efforts" [5]) for 1992 through 1993 that was based on state cigarette prices and smoking bans at both workplace and home (2). This index correlated with decreases in both smoking prevalence (among adults aged ≥25 years) and tobacco consumption (all ages) by state starting in 1993 (2). For adults aged 30 to 39 years, the IOI was inversely correlated with prevalence of current smoking, positively correlated with proportion of ever smokers who had quit, and negatively correlated with lung cancer death rates for adults aged 30 to 39 years in 33 states (5).

Trends in tobacco-related cancers other than lung cancer have received little attention. For bladder cancer incidence trends from 1975 through 1999 in 9 cancer registries in the National Cancer Institute's Surveillance, Epidemiology and End Results (SEER) Program, a model of incidence (with a 3-year lag) produced a negative regression coefficient (bTCP or coefficient associated with the Tobacco Control Program in San Francisco-Oakland, California) that approached significance (bTCP = –0.235, *P* = .07 for trend in bladder cancer rate in San Francisco-Oakland). However, the other SEER areas of California were not included (7). Trends in oral cavity-pharynx cancers are also important, because tobacco interacts strongly (and multiplicatively) with alcohol use, greatly increasing risk.

Low socioeconomic status (SES) has generally been associated with higher risks of tobacco-related cancers, probably reflecting (in part) differences in smoking habits (8). Data from national surveys have shown that smoking and successful or long-term quitting among smokers are strongly related to SES indicators (9,10).

Trends in incidence rates since 1992 were examined for lung, oral cavity-pharynx, and bladder cancers by SEER geographic areas, which had populations differing in history of state tobacco control efforts and an SES indicator.

## Methods

Adults aged 15 to 54 years were the youngest age group with statistically reliable data for temporal trends by geographic area for invasive (malignant) cancers in each of the 3 categories (oral cavity-pharynx, lung-bronchus, and bladder). For bladder cancer, "in situ" cancers had been recoded to "invasive" in SEER databases because of inaccuracies in differentiating these cancers (11,12). Declines in risks for all of the cancers studied begin within only a few years of quitting smoking (9). Incidence rates for other smoking-related cancers (eg, esophageal and laryngeal cancers) among the population aged 15 to 54 years were too low for meaningful analyses of trends, whereas mortality rates (available for all states) are lower and are influenced by survival rates.

Age-standardized incidence rates (ASIRs) for the population aged 15 to 54 years for lung-bronchus, oral cavity-pharynx, and bladder cancers from 1992 through 2004 were analyzed for each of 11 SEER areas: the metropolitan areas of Atlanta, Georgia, and Detroit, Michigan; Connecticut; Hawaii; Iowa; New Mexico; Utah; Seattle-Puget Sound, Washington; and Los Angeles County, San Francisco-Oakland, and San Jose-Monterey in California (11). SEER data are the only resource for analysis of long-term cancer incidence trends. For the 3 areas of California, data are available starting with diagnoses in 1992, and 2004 was the latest year for which incidence data were complete (11,12). ASIRs for the 2 other SEER areas (rural Georgia and Alaska) were too statistically unreliable for analysis of annual percentage change (APC) (11,12) but were included in analysis of all 13 SEER areas combined.

The poverty rate from the 1990 census, strongly correlated with other measures of SES, has been used in previous analyses of SEER data, by county and other geopolitical units, to measure economic deprivation and uneven distribution of economic resources in a population that is (13). Cancer incidence trends were tabulated for each SEER area, ranked from lowest to highest poverty rates (from the 1990 census) (14) for the white and black populations of each SEER area.

The 11 SEER areas involved states with different histories of tobacco control efforts, as measured by the IOI for 1992 through 1993 (2), based on the sum of *z* scores on state cigarette price and (from the Current Population Survey) percentages of homes and workplaces with restrictions on smoking. Negative values indicate states below the median (2,5). California, Connecticut, Hawaii, Utah, and Washington ranked among the top 10 on this IOI. New Mexico ranked 19th; Georgia (a tobacco-growing state) ranked 31st; Iowa, 37th; and Michigan, 40th (2,5), Table 1. This IOI was constructed before full implementation of the American Stop Smoking Intervention Study (ASSIST) (5). Another IOI, defined in a 2006 ASSIST report (15), was based on cigarette prices, a rating of local and state clean indoor air policies, and the percentage of workers covered by 100% smoke-free workplaces (15) for 1992 through 1993 and 1998 through 1999 (Table 1). The strength of tobacco control (SoTC) index, which comprises variables for state resources (staff and funds committed to tobacco control), state capacity (infrastructure to deliver state-level tobacco control), and program efforts (focused on policy and socioenvironmental change) was calculated only for 1999 through 2000 (15).

For the 11 SEER areas, the original IOI for 1992 through 1993 was strongly correlated with the revised IOI for 1992 through 1993 (Pearson *r* = .908, *P* < .001) and with the IOI for 1998 through 1999 (15) (*r* = .678, *P* = .02) but less strongly with the SoTC for 1999 through 2000 (*r* = .468, *P* = .15) (data not tabulated). For the SoTC, California's high score (+3.73) stands out among the SEER states (Table 1); among all 50 states, only Arizona had a higher SoTC (+4.03) (15). For each index in Table 1, the value for California was assigned to each of the 3 SEER areas in California under the assumption that statewide measures of tobacco control were equally applicable to each of these areas.

APC in the ASIR was available for 1992 through 2004; 1992 corresponds to a time before any impact of state tobacco control efforts measured for 1992 through 1993 would be expected on ASIRs, although state tobacco control efforts began before 1992 and California's comprehensive program was begun in 1989 (2,5). The APC was calculated by using weighted least squares regression of natural logarithms of ASIRs (11,12); standard errors and confidence limits (CL) (Tiwari method) were calculated from the fitted regression, assuming a constant rate of change by calendar year (11,12).

APCs were calculated for whites and (where possible) blacks. For blacks, 5 SEER areas had a black population greater than 250,000 in the 1990 census; other SEER areas had black populations less than 150,000 (<50,000 for most) in 1990. APCs for blacks are tabulated only for the 5 SEER areas for which APCs could be calculated for both lung and oral cavity-pharynx cancers; the output from SEER Program software (SEER*Stat, National Cancer Institute, Bethesda, Maryland) (11) indicates which APCs cannot be calculated because of the statistical instability of ASIRs. ASIRs and APCs were very similar for all whites and non-Hispanic whites in each of the SEER areas so that only data for all whites are tabulated. Other racial/ethnic groups (eg, Asian Americans and Pacific Islanders) comprise small populations in most SEER areas (other than California), and APC could not be calculated.

In addition to tabulations of APCs for 1992 through 2004, we plotted ASIRs for individual calendar years for selected SEER areas with large APCs to examine consistency in trends over time (relevant to the linear assumption involved in calculating CLs for APCs) (11,12) and to assess the potential impact of delayed reporting of cancers to central (SEER) cancer registries, which should affect mainly the latest year of diagnosis covered (2004 in this study) (12).

Correlation coefficients (Pearson *r'*s) were calculated by using SPSS version 15.0 (SPSS Inc, Chicago, Illinois) between the APCs for each cancer-site group by SEER area and the state's poverty rate (1990 census) and with the state's score on the selected indices of tobacco control efforts (Table 1).

## Results

Among whites, the largest negative APCs (−5% to −6%) for lung cancer were in the 3 California SEER areas, and 95% confidence intervals (CIs) for these APCs did not overlap with CIs for most of the other SEER areas. Although the negative APC for lung cancer in Iowa, which had a white poverty rate of 10% in 1990, was significant, it was only −1.3%; Utah and New Mexico also had low APCs (−1.1% and −1.2%). These 3 areas had relatively high poverty rates among whites in 1990 (>10%, Table 2). For oral cavity-pharynx cancers in whites, only the 3 California SEER areas had significant negative APCs, and Utah was the only other area with a negative (albeit not significant) APC (Table 2). For bladder cancer among whites, APCs were negative for all areas except San Jose-Monterey and Utah and were significant for a few areas (San Francisco-Oakland, Connecticut, and Detroit), all with relatively low poverty rates for whites (4%-6%). San Francisco-Oakland was the only California area with a significant negative APC for bladder cancer (Table 2).

For whites, the poverty rate of each SEER area (1990 census) was strongly correlated with the APC for lung cancer (but did not reach significance for the sample size of 11 geographic areas) and weakly correlated with the APCs for oral cavity-pharynx or bladder cancer (Table 3). For whites, the strongest correlations between the APCs and the tobacco control indexes were for the SoTC with both lung cancer and oral cavity-pharynx cancers (but not bladder cancer) (Table 1). The SoTC was not strongly correlated with the poverty rate for whites by SEER area (*r* = −.311, *P* = .35). The other indices of state tobacco control efforts were also negatively correlated with APCs for lung and oral cavity-pharynx, but not bladder; only the correlations with oral cavity-pharynx APCs reached significance (for the original IOI for 1992 through 1993 and the revised IOI for 1992 through 1993 but not the IOI for 1998 through 1999) (Table 3). The stronger correlation of IOIs with oral cavity-pharynx than lung cancer (Table 3) reflects the large negative APCs for lung cancer in many SEER areas compared with the restriction of large negative APCs for oral cavity-pharynx to the 3 California areas (Table 2), and the strong correlations of APCs for lung and oral cavity-pharynx with the SoTC reflect the uniquely high SoTC for California and the large negative APCs for these cancers in the 3 California SEER areas (Table 2).

ASIRs for lung and oral-cavity cancer (but not bladder cancer) were higher for blacks than for whites. Among blacks, all but a few of the SEER regions with useful data on APCs had significant negative APCs for both lung and oral cavity-pharynx cancers. For bladder cancer as well as the other 2 cancer-site groups among blacks, Detroit had a significant (negative) APC, despite the high poverty rate for the black population (Table 2). The small number of SEER areas with useful data on APCs for blacks and the general uniformity of APCs across SEER areas precluded meaningful analyses of correlation coefficients between APCs for blacks and the tobacco control indices by SEER area.

Analyses by sex were limited to whites (data not tabulated) because of statistically unstable ASIRs for blacks aged 15 to 54 years. Significant negative APCs for lung cancer in the 3 California SEER areas were found for both men and women. For oral cavity-pharynx cancers, 4 of the 6 negative APCs in the 3 California areas were significant but only 1 other APC was significant (that for Utah men). For bladder cancer, only men had a significant negative APC (2 in California, along with Connecticut, Atlanta, and Iowa), but in Detroit APCs approached significance for both men (−2.4, 95% CL = −4.9 to 0) and women (−2.9, 95% CL = −5.6 to 0).

ASIRs for the population aged 15 to 54 years for all 13 SEER areas combined showed large declines for lung-bronchus cancer among both whites and blacks from 1992 to 2004, a larger decline among blacks than whites for oral cavity-pharynx cancer, and only small declines for bladder cancer among both blacks and whites (Table 2). For the 13 areas combined, ASIRs in 2004 were still higher for lung and oral cavity-pharynx cancer and lower for bladder cancer among blacks than whites, and 95% CIs did not overlap for the 2 groups (although they were closer for oral cavity-pharynx cancer in 2004 than in 1992) (Table 2).

**Figure 1:**
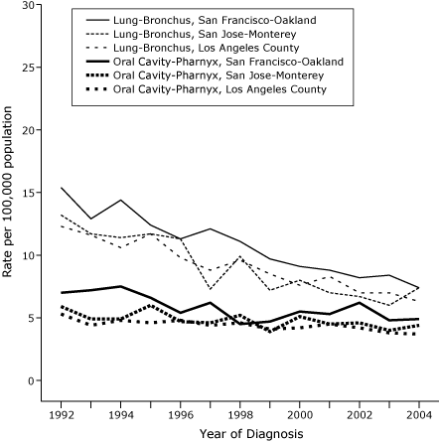
Age-standardized incidence rates per 100,000 population per year for lung-bronchus and oral cavity-pharynx cancers in white populations aged 15 to 54 years, 3 California areas in the Surveillance, Epidemiology and End Results (SEER) Program, by year of diagnosis, 1992-2004.

**Figure 2:**
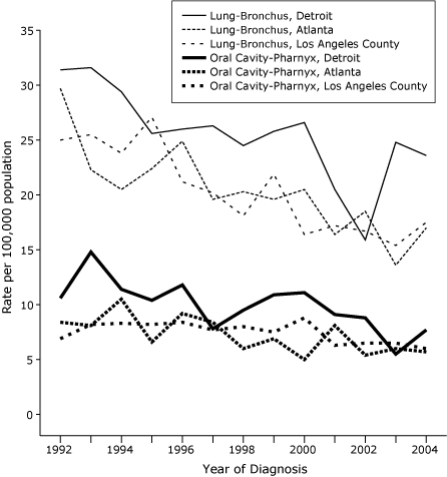
Age-standardized incidence rates per 100,000 population per year for lung-bronchus and oral cavity-pharynx cancers in black populations aged 15 to 54 years, 3 areas in the Surveillance, Epidemiology and End Results (SEER) Program, by year of diagnosis, 1992-2004.

ASIRs for each year of diagnosis from 1992 through 2004 are shown in Figure 1 for whites aged 15 to 54 years in each of the 3 SEER areas (all in California) that had significantly negative APCs for both lung and oral cavity-pharynx cancer; in each area, ASIRs for lung cancer converged toward those for oral cavity-pharynx cancers. For SEER areas with the most statistically reliable data for blacks, declines in APCs were evident for lung and oral cavity-pharynx cancers in blacks, including Detroit (Figure 2). Declines were generally continuous, with some fluctuations from year to year but no indication that a recent downturn (eg, due to delayed reporting of cancers to SEER registries) was responsible for the negative APCs (Figures 1 and 2).

**Table:** 

**Year**	**Lung-Bronchus, San Francisco-Oakland**	**Lung-Bronchus, San Jose-Monterey**	**Lung-Bronchus, Los Angeles County**	**Oral Cavity-Pharynx, San Francisco**	**Oral Cavity-Pharynx, San Jose**	**Oral Cavity-Pharynx, Los Angeles County**
1992	15.4	13.2	12.3	7.0	5.9	5.3
1993	12.9	11.7	11.6	7.2	4.9	4.4
1994	14.4	11.4	10.6	7.5	4.9	4.8
1995	12.4	11.7	11.7	6.6	6.0	4.6
1996	11.3	11.3	9.8	5.4	4.7	4.8
1997	12.1	7.3	8.8	6.2	4.6	4.4
1998	11.1	9.9	9.6	4.5	5.2	4.6
1999	9.7	7.2	8.5	4.7	3.9	4.1
2000	9.1	8.0	7.6	5.5	5.1	4.2
2001	8.8	7.0	8.3	5.3	4.5	4.5
2002	8.2	6.7	7.0	6.2	4.6	4.2
2003	8.4	6.0	7.0	4.8	4.0	3.8
2004	7.4	7.4	6.3	4.9	4.4	3.7

**Table:** 

**Year**	**Lung-Bronchus, Detroit**	**Lung-Bronchus, Atlanta**	**Lung-Bronchus, Los Angeles County**	**Oral Cavity-Pharynx, Detroit**	**Oral Cavity-Pharynx, Atlanta**	**Oral Cavity-Pharynx, Los Angeles County**
1992	31.4	29.7	25.0	10.6	8.4	6.9
1993	31.6	22.3	25.5	14.8	8.1	8.2
1994	29.4	20.5	23.8	11.4	10.5	8.3
1995	25.6	22.4	27.1	10.4	6.6	8.2
1996	26.0	24.9	21.2	11.8	9.2	8.4
1997	26.3	19.6	20.2	7.8	8.4	7.7
1998	24.5	20.3	18.1	9.5	6.0	8.0
1999	25.8	19.6	21.9	10.9	6.9	7.5
2000	26.6	20.5	16.4	11.1	5.0	8.8
2001	20.5	16.4	17.2	9.1	8.1	6.3
2002	15.9	18.5	16.7	8.8	5.4	6.5
2003	24.8	13.6	15.4	5.5	6.0	6.5
2004	23.6	17.0	17.5	7.7	5.7	6.0

## Discussion

The smaller declines in ASIRs for the population aged 15 to 54 years for the cancers most strongly associated with smoking (ie, lung-bronchus and oral cavity-pharynx) among whites in the 3 SEER areas with the highest white poverty rates (Iowa, New Mexico, and Utah) (10%-14%) in 1990 than in most other areas could suggest an SES effect on trends, although ASIRs for lung cancer were already low in 1992 in New Mexico and Utah (Table 2), and the correlation coefficients between poverty rate and APCs did not reach significance for the numbers of areas available for analysis. However, for whites, the largest negative APCs for both lung and oral cavity-pharynx cancers were in the 3 California SEER areas, despite a white poverty rate of 9% in Los Angeles County (Table 2).

Although state tobacco control efforts have been measured by various indices and have varied over time, the most striking findings are the larger declines in APCs for lung and oral cavity-pharynx cancers for whites in the 3 SEER areas of California, and the strong correlations between these APCs and both the IOIs and especially the SoTC for 1999 through 2000. These findings are also noteworthy in view of declines in cigarette consumption (16,17) and in prevalence of current smokers among younger adults in California (18). Successful quit ratios among smokers aged 20 to 49 years from 1992 to 2002 were higher in California than in comparison states where state tobacco control efforts were more limited (3). These states include New York and New Jersey combined and 6 southern states combined (3) (which had the lowest quit rate). Data on estimated cigarette smoking prevalence in adults for selected metropolitan areas and counties in the United States (including those in the SEER program) from the Behavioral Risk Factor Surveillance System surveys became available only starting with the 2002 survey (19), so temporal trends cannot be examined.

The convergence of ASIRs for lung cancer among whites in each of the 3 California SEER areas toward the ASIRs for oral cavity-pharynx cancer (Figure 1) may reflect the higher smoking-attributable fraction for lung cancer (9). Negative APCs for lung-bronchus and oral cavity-pharynx cancers in the 3 California SEER areas were larger than those in certain other SEER areas (Seattle-Puget Sound, Utah, Hawaii, and Connecticut) that also ranked high on a state tobacco control index for 1992 through 1993 (2). This finding suggests the importance of factors specific to California. California had the earliest comprehensive program, and its per capita spending on tobacco control was high in the 1990s (3), although it was later surpassed by other states. Among the 11 SEER areas, a high SoTC for 1999 through 2000 was unique to California (Table 1). Data are needed, however, on cancer incidence trends in other (non-SEER) states with high SoTCs (15).

A decline that approached significance was seen in ASIRs for bladder cancer among whites of all ages in the San Francisco-Oakland SEER area from 1975 through 1999 (7). Current findings for whites aged 15 to 54 show a significant negative APC in San Francisco-Oakland but not in the other 2 California areas. This finding may reflect the lower attributable fraction of bladder cancer (due to smoking) compared with that for lung-bronchus and oral cavity-pharynx cancers (9,20). Among whites aged 35 to 64 years, estimated smoking-attributable fractions for women are 32% for bladder cancer, 77% for lung-bronchus cancer, and 55% for oral cavity-pharynx cancer (19). For men the rates are 48%, 89%, and 77%, respectively. For lung and oral cavity-pharynx cancers, geographic variation in other cancer risk factors such as fruit and vegetable and alcohol consumption also may be involved, but the causes of bladder cancer are poorly understood, and attributable fractions (for known risk factors) may vary geographically (21).

For reasons that are not completely understood, blacks have higher ASIRs for lung-bronchus and oral cavity-pharynx cancers but lower ASIRs for bladder cancer (14). Among the limited number of SEER areas with useful data for lung-bronchus and oral cavity-pharynx cancers in blacks (Table 1, Figure 2), the generally uniform (negative) APCs are noteworthy in view of uniformly large declines in smoking prevalence among blacks aged 20 to 64 years from 1992 through 1993 and 2001 through 2002 in several states where cigarette prices and tobacco control efforts differed, including California, New York, and New Jersey combined, and 6 southern tobacco-growing states combined (18). Part of the explanation for these findings may be that trends in smoking initiation have been involved in the disappearance of black-white disparities in US smoking prevalence, but further research is needed on the impact of tobacco control efforts in black populations (18).

Study limitations include the problematic interpretation of temporal trends in ASIRs. The large declines in lung and oral cavity-pharynx cancers in California could have occurred even in the absence of statewide tobacco control efforts that started in 1989. SEER data on ASIRs before 1992 in the California SEER areas are available for San Francisco-Oakland (11), however, and only small changes in ASIRs for whites occurred from 1973 to 1991 for lung (from 20.1 to 17.3 per 100,000) and oral cavity-pharynx cancers (from 8.5 to 8.7) (data not tabulated), compared with the large declines from 1992 through 2004.

Another study limitation is that only a limited number of geographic areas could be considered in the analysis of APCs in ASIRs from 1992 through 2004, including only the 3 SEER areas in California and not the entire state. Trends in ASIRs for lung cancer for all ages combined in California reported for 1988 through 1997 and compared with non-California SEER data (22) should be updated to include analysis of lung and other tobacco-related cancers in younger adults by race/ethnicity. Starting with cancer diagnoses in 2000, ASIRs for tobacco-related cancers by age group can be compared for 39 states with high-quality cancer data (23) that differ by socioeconomic indicators and history of statewide tobacco control efforts, including Arizona (with the highest SoTC for 1999 through 2000) (15) and states with large black populations.

Despite the limitations of ecologic analyses and their interpretation, the findings for whites in the 3 California SEER areas could provide impetus for expansion of state tobacco control efforts in other states, along with the evidence that California experienced significantly larger temporal increases in smoking cessation rates among smokers younger than 35 years than did several comparison states (3). Future changes in tobacco-related cancer incidence rates should reflect the effect of tobacco control programs on smoking initiation (as well as smoking cessation), as youths prevented from adopting the smoking habit reach the ages at which tobacco-related cancer incidence rates rise sharply (24). If California's smoking initiation and cessation rates could be attained nationally, a target of 14% smoking prevalence by 2020 has been suggested as feasible (25), and reductions in incidence rates for tobacco-related cancers (as well as other diseases) should ensue. Increasing cigarette prices by states may have become a less effective tool for reducing smoking prevalence among low-income smokers after the Master Settlement Agreement of 1998 (26), but the need for comprehensive prevention and cessation programs in "those populations paying the greatest share of the increased prices" has long been recognized (27).

## Figures and Tables

**Table 1:** Indices^a^ of State Tobacco Control Efforts: Initial Outcomes Index (IOI) and Strength of Tobacco Control (SoTC) Index, SEER Program Areas

**State^b^**	IOI-1 1992-1993^c^	IOI-2 1992-1993^d^	IOI-2 1998-1999^e^	SoTC 1999-2000
Washington (Seattle)	+4.62	+4.40	+8.45	+0.23
California (3 areas)^f^	+4.62	+4.25	+6.74	+3.73
Utah	+4.01	+3.68	+7.77	−0.29
Hawaii	+3.21	+3.10	+9.04	+0.96
Connecticut	+2.34	+0.44	+3.47	+0.37
Georgia (Atlanta)	−0.49	−1.87	+1.73	+0.39
Michigan (Detroit)	−1.59	+0.76	+6.64	+0.90
Iowa	−1.18	−1.24	+2.17	+0.41
New Mexico	+1.05	+0.17	+2.70	−0.53

Abbreviations: SEER, Surveillance, Epidemiology and End Results.

a Indices are based on sums of standardized scores for each component; negative values indicate states below the median for all 50 states plus the District of Columbia (2,5,15). For metropolitan areas in the SEER program, the statewide index was assigned (eg, the same value for each of the 3 SEER areas in California).

b States are ranked from highest to lowest on an IOI for 1992-1993 (2,5).

c IOI-1 indicates version 1 for 1992-1993 (2,5).

d IOI-2 indicates version 2 for 1992-1993 (15).

e IOI-2 indicates version 2 for 1998-1999 (15).

f Los Angeles County, San Francisco-Oakland, and San Jose-Monterey.

**Table 2:** Annual Percentage Change^a^ in Age-Standardized Incidence Rate for Invasive Lung-Bronchus, Oral Cavity-Pharynx, and Bladder Cancers in Whites and Blacks Aged 15 to 54 Years, SEER Program Areas, 1992 and 2004

Area^b^	Poverty Rate, %^c^ valign="bottom"	Age-Standardized Incidence Rate (per 100,000)		Annual Percentage Change, % (95% CI)

		1992	2004	
**Lung-Bronchus**				
**White**				
Connecticut	4	16.0	11.8	−2.6 (−3.2 to −2.9)
Atlanta	5	15.0	9.8	−4.1 (−4.9 to −3.2)
San Francisco-Oakland	6	15.4	7.4	−5.6 (−6.5 to −4.8)
San Jose-Monterey	6	13.2	7.4	−6.0 (−7.8 to −4.2)
Detroit	6	18.1	13.4	−2.4 (−3.5 to −1.2)
Seattle-Puget Sound	7	15.1	10.2	−2.7 (−3.9 to −1.5)
Hawaii	7	18.0	9.0	−3.4 (−5.9 to −0.9)
Los Angeles County	9	12.3	6.3	−5.2 (−6.1 to −4.3)
Iowa	10	15.0	12.9	−1.3 (−2.5 to −0.1)
Utah	10	6.2	5.3	−1.1 (−3.9 to 1.9)
New Mexico	14	8.4	7.4	−1.2 (−3.3 to 0.9)
13 areas	NA	14.1	9.2	−3.4 (−3.8 to −3.1)
CI	NA	13.5 to 14.8	8.7 to 9.6	NA
**Black^d^**				
Connecticut	16	24.0	12.3	−4.3 (−7.4 to −1.2)
San Francisco-Oakland	17	32.6	19.7	−4.7 (−6.4 to −2.9)
Los Angeles County	17	25.0	17.5	−4.2 (−5.7 to −2.8)
Atlanta	18	29.7	17.0	−4.2 (−5.7 to −2.8)
Detroit	27	31.4	23.6	−3.0 (−4.9 to −1.0)
13 areas	NA	28.8	17.7	−4.2 (−4.8 to −3.4)
CI	NA	26.2 to 31.6	16.1 to 29.4	NA
**Oral Cavity-Pharynx**				
**White**				
Connecticut	4	4.8	5.0	−0.1 (−2.8 to 1.6)
Atlanta	5	4.9	5.5	0.2 (−2.7 to 3.2)
San Francisco-Oakland	6	7.0	4.9	−3.2 (−5.9 to −1.3)
San Jose-Monterey	6	5.9	4.4	−2.1 (−3.7 to −0.4)
Detroit	6	5.6	4.8	0.6 (−1.5 to 2.7)
Seattle-Puget Sound	7	5.3	6.2	0.3 (−1.6 to 2.3)
Hawaii	7	7.0	10.6	0.1 (−3.2 to 3.6)
Los Angeles County	9	5.3	3.7	−2.2 (−3.1 to −1.2)
Iowa	10	4.6	5.1	1.0 (−0.5 to 2.5)
Utah	10	4.8	3.3	−1.9 (−4.3 to 0.5)
New Mexico	14	4.8	3.3	0.3 (−3.4 to 4.1)
13 areas	NA	5.4	4.8	−0.8 (−1.6 to 0)
CI	NA	5.0 to 5.8	4.4 to 5.1	NA
**Black^d^**				
Connecticut	16	21.2	4.8	−11.4 (−16.3 to −6.3)
San Francisco-Oakland	17	4.6	8.1	−2.0 (−6.8 to 3.1)
Los Angeles County	17	6.9	6.0	−2.0 (−3.7 to −0.3)
Atlanta	18	8.4	5.7	−4.0 (−6.8 to −1.2)
Detroit	27	10.6	7.7	−4.1 (−5.0 to −2.7)
13 areas	NA	8.9	6.2	−3.8 (−5.0 to −2.7)
CI	NA	7.5 to 10.4	5.3 to 7.2	NA
**Bladder**				
**White**				
Connecticut	4	6.4	4.8	−2.9 (−4.7 to −1.2)
Atlanta	5	5.3	4.3	−2.4 (−5.0 to −0.1)
San Francisco-Oakland	6	4.9	2.6	−2.7 (−5.3 to −0.1)
San Jose-Monterey	6	3.4	4.2	1.0 (−2.5 to 4.7)
Detroit	6	6.2	4.1	−2.5 (−4.4 to −0.6)
Seattle-Puget Sound	7	4.9	4.3	−0.8 (−3.4 to 1.8)
Hawaii	7	4.6	4.1	−1.0 (−4.3 to 2.3)
Los Angeles County	9	3.8	2.8	−1.4 (−2.0 to 0)
Iowa	10	5.0	3.8	−1.2 (−2.7 to 0.4)
Utah	10	2.4	2.9	0 (−2.3 to 2.3)
New Mexico	14	3.9	2.7	−2.2 (−5.6 to 1.2)
13 areas	NA	4.7	3.6	−1.8 (−2.6 to −1.0)
CI	NA	4.3 to 5.1	3.3 to 3.9	NA
**Black^d^**				
Connecticut	16	e	e	e
San Francisco-Oakland	17	e	e	e
Los Angeles County	17	2.5	2.4	0.3 (−5.0 to 5.9)
Atlanta	18	1.5	1.4	−1.6 (−6.6 to 3.6)
Detroit	27	2.9	1.9	−3.1 (−5.0 to −0.5)
13 areas	NA	2.5	2.1	−1.3 (−3.3 to 0.8)
CI	NA	1.8 to 3.4	1.6 to 2.7	NA

Abbreviations: SEER, Surveillance, Epidemiology and End Results; CI, confidence interval; NA, not applicable.

a The annual percentage change was calculated using weighted least-squares regression of natural logarithms of age-standardized incidence rates (11,12).

b Program areas are ranked from lowest to highest according to poverty rate, by race.

c Poverty rate represents each SEER area for each race (white or black) from the 1990 census (14).

d SEER areas had statistically unstable age-standardized incidence rates, and annual percentage changes could not be calculated for any of the 3 types of cancer in Hawaii, Iowa, New Mexico, Seattle-Puget Sound, Utah, and San Jose-Monterey (11).

e Age-standardized incidence rates showed especially large fluctuations from year to year.

**Table 3:** Correlation Coefficients for State Tobacco Control Indices and Annual Percentage Change in Cancer Incidence Rates From 1992 to 2004 in the White Population Aged 15 to 54 Years, Selected SEER Program Areas

Index/Poverty Rate	Lung-Bronchus		Oral Cavity-Pharynx		Bladder	

	Pearson *r*	*P* Value	Pearson *r*	*P* Value	Pearson *r*	***P* Value**
IOI-1^a^, 1992-1993	−0.46	.15	−0.69	.02	0.46	.15
IOI-2^b^, 1992-1993	−0.47	.15	−0.71	.01	0.51	.11
IOI-2^c^, 1998-1999	−0.26	.44	−0.41	.21	0.46	.15
SoTC, 1999-2000	−0.96	<.001	−0.75	.007	0.18	.60
Poverty rate, 1990	0.52	.10	0.10	.77	0.17	.61

Abbreviations: SEER, Surveillance, Epidemiology and End Results; IOI, Initial Outcomes Index (2,5,15); SoTC, Strength of Tobacco Control (15).

a IOI-1 indicates version 1 for 1992-1993 (2,5).

b IOI-2 indicates version 2 for 1992-1993 (15).

c IOI-2 indicates version 2 for 1998-1999 (15).
